# Supplementary material for: Clinical Validation of Digital Healthcare Solutions: State of the Art, Challenges and Opportunities
Source: Healthcare (Basel). 2024 May 22;12(11):1057. doi: 10.3390/healthcare12111057 (PMC11171879; doi:10.3390/healthcare12111057)
Supplement: Supplementary file 1 [file healthcare-12-01057-s001.zip › healthcare-2963345-supplementary.pdf]

Figure S1. Categorisation of the articles included in the review by topic.

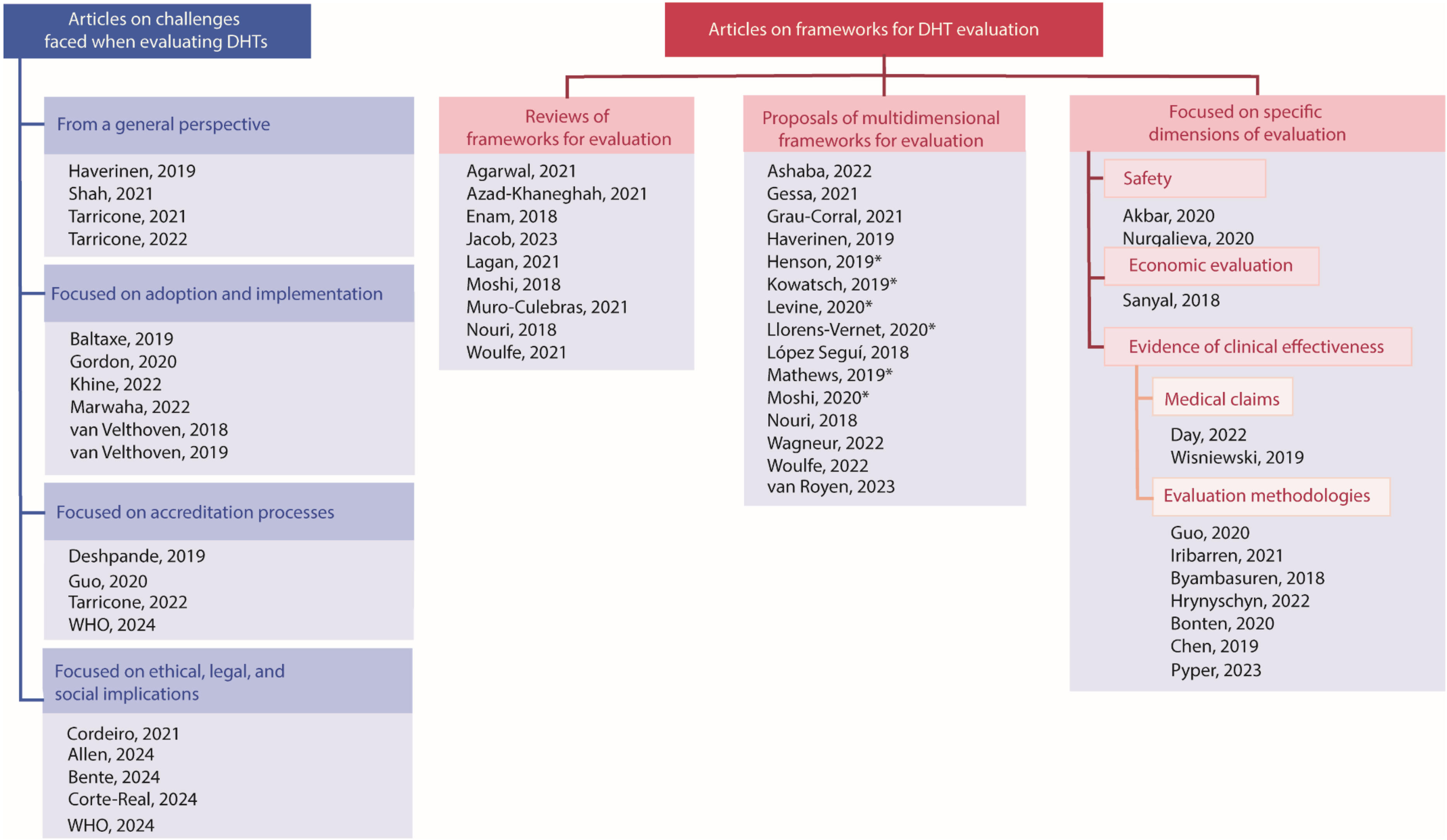

*Already included in any of the examined reviews of frameworks.

Table S1. List of terms in Abstract/Keywords/Title according to dimension or scope of application.

Dimension	term
Usability	Usability
	Usability testing
	User experience
	User satisfaction assessment
	Human-computer interaction
	User acceptance testing
	User feedback analysis
	Satisfaction
	User interface evaluation
	Ease of use assessment
	Learnability evaluation
	Accessibility testing
	User friendliness
	User engagement
	Acceptability evaluation
	Task efficiency assessment
	User-perceived value
	Accessibility
Security	Security
	Security and privacy
	Privacy
	Clinical safety
	Safety
Feasibility	Feasibility
	Proof of concept
	Proof-of-concept
	Prototype Test
	Prototype-Test
	Concept Validation
Functionality	Functionality
Effectiveness	Effectiveness
Efficacy	Efficacy
Efficiency	Efficiency
	Cost-effectivity

	Cost-effectiveness
	Cost-effective
	Cost-utility
	Cost effectivity
	Cost effectiveness
	Cost effective
	Cost utility
	Economic impact
Implementation	Implementation study
	Standard of care
	Adoption
	hype cycle

Table S2. Details of the reviewed publications proposing a multidimensional evaluation framework or model.

Reference author, year	DHT category	Scope (target)	Development	Framework/models evaluation: dimensions/criteria/steps for assessment
**Ashaba, 2022**	General	Framework for design and evaluation (eHealth implementers in developing countries)	Experts	**Based on recommendations of eHealth evaluation literature, with 2 dimensions and the corresponding performance indicators.**<br>• **Monitoring:** technological, organisational, and legal and ethical aspects.<br>• **Evaluation:** eHealth outputs (human/social and user coverage and transferability aspects), eHealth outcomes (efficiency, quality, utilisation and cost aspects), and impact (disease prevalence and incidence, mortality rates, QoL, and health equity).
**Gessa, 2021**	mHealth	Assess DHT quality (governments, clinicians, and health researchers)	Researchers	**Based on a set of accredited apps, a classification framework comparing verified quality (certification program requirements) and perceived quality (user experiences):**<br>• **Accredited quality:** degree of compliance with the accreditation standard requirements [design and appropriateness (appropriateness, accessibility, design, and usability/testing), quality and safety of information (suitability for the audience, transparency, authorship, information updates/revisions, content and information sources, and risk management), provision of services (technical support/inquiries, E-commerce, bandwidth, and advertisement), and confidentiality and privacy (privacy and data protection, and logical security] grouped into 4 categories: **design and relevance, quality and safety, services, and confidentiality and privacy.**<br>• **Perceived quality by the users:** % of positive comments/total comments grouped in 4 quartiles.<br>• **Perceived quality vs verified quality assessed through crosstabs.**
**Grau-Corral, 2021**	mHealth	Assess quality for DHT informed selection (HCPs)	Experts panel	**Based on literature review, development of the 17-items ISYScore-Pro scale within 3 dimensions:**<br>• **Trust:** Validated by a health agency, scientific society, health care professional college, or nongovernmental organisation; Authors are explicitly identified; Website is accessible (responsibility); Cites peer-reviewed sources; Names the organisation responsible; Updated within the last calendar year; Disclosure on how the app was financed.<br>• **Utility:** increases capacity (Provides calculations and measurements; Helps in a care procedure; Archives data images); increases the experience (Facilitates observation of cause-effect relationships and allows users to rehearse; Facilitates observation of those who do well (vicarious learning); and Facilitates patient follow-up); increases social relationships (Obtains positive feedback and Provides social content).<br>• **Interest:** Positive user ratings/downloads and content available in other formats (e.g., web, tablet, or magazines).

Reference author, year	DHT category	Scope (target)	Development	Framework/models evaluation: dimensions/criteria/steps for assessment
**Haverinen, 2019**	mHealth, AI, and robotics	Development of an HTA framework (decision makers)	Interviews with technology companies and healthcare service providers and expert's workshops	**Digi-HTA: 11 domains needed to be included in HTA frameworks for digital health (apart from the traditional ethical, social, and legal issues):**<br>• **Company information:** 3 questions (contact information of company, business model, and whether there are management systems in use).<br>• **Product information:** 22 questions (e.g., platforms and versions available, plans for pots-marketing surveillance, intended user groups, or whether it needs user's training).<br>• **Cost:** 6 questions (costs for a healthcare consumer, maintenance costs, or how often it has to be renewed)<br>• **Effectiveness:** 6 questions (e.g., What kind of evidence is available for effectiveness (case studies, randomized controlled trials, Cochrane reviews, etc.)? Are there any ongoing studies to investigate the product's effectiveness?)<br>• **Clinical safety:** 9 questions (e.g., whether there are risks, possible side effects associated with the use, responsible person in the company for handling incident reports).<br>• **Technical stability:** 6 questions (e.g., company's testing process, capacity to go to back to previous versions, or capacity to monitor the system)<br>• **Usability and accessibility:**<br>• **Interoperability:** 11 questions (e.g., existence of interfaces into the website, other software, or other companies' services).<br>• **Data security and protection:** to be prepared based in criteria defined in separate documents.<br>• **AI:** 20 questions (e.g., define the problem to be solved, whether it could be solved without AI, what are the data sources, whether retraining is possible, how many tests are needed for the model, or whether changes the care process).<br>• **Robotics:** 4 questions (e.g., whether it creates safety risks for the healthcare personnel).
**Henson, 2019***	mHealth	Framework for quality evaluation (patients and clinicians)	Clinicians, patients, family members, researchers and policy-makers	Assessment criteria from a **systematic review of frameworks to create a new framework with 5 priority levels and their corresponding subcategories** (users should begin at the bottom or level 1 and do not need to proceed if any single level does not meet their needs).<br>• **Level 1. Background info:** business model, technical, credibility, cost and advertising, medical claims, and stability.<br>• **Level 2. Privacy and security:** privacy policy, data storage, data collected, personal health information, security measures in place, and deleting personal data.<br>• **Level 3. Evidence based:** first impressions, impressions after using, clinical validity, and user feedback supporting.<br>• **Level 4. Easy of use.** Short-term usability, long-term usability, and specificity to users and accessibility.<br>• **Level 5. Data integration.** Data ownership, access an export, clinically actionable, and therapeutic alliance.

Reference author, year	DHT category	Scope (target)	Development	Framework/models evaluation: dimensions/criteria/steps for assessment
**Kowatsch, 2019***	General	Framework for design and evaluation (researchers and practitioners)	Researchers	**DEDHI life cycle framework based on systematic review of evaluation criteria and implementation barriers.**<br>• **Preparation phase.** The ***goals and tasks*** are to define the conceptual and technological foundation of a DHI. The ***technical maturity*** refers to the research prototype that provides basic functionality to assess the feasibility of the DHI. ***Evaluation criteria***: Ease of use, adherence, personalization, safety, and privacy and security. ***Implementation barriers*** include social interaction, individual characteristics of end user, usability (e. g., user-centred design), expectations (privacy, confidentiality, security), negative associations, workforce, planning, funding (no funding), cost (start-up), standards, and regulatory issues.<br>• **Optimization phase:** The ***goals and tasks*** are to build an optimized DHI by selecting effective intervention components. The ***technical maturity*** refers to an elaborated research prototype that provides the full functionality according to the conceptual model to assess the health impact of DHI components. ***Evaluation criteria:*** effectiveness (individual components of the DHI), perceived benefit, content quality, personalization, perceived enjoyment, aesthetics, adherence, service quality, safety, and privacy and security***. Implementation barriers*** include social support, outcome expectations, usability, funding (for equipment), cost (of technology), and integration.<br>• **Evaluation phase:** The ***goals and tasks*** are to confirm the effectiveness of an optimized DHI. The ***technical maturity*** refers to an elaborated research prototype that provides the full functionality according to the conceptual model to assess the effectiveness of the DHI. ***Evaluation criteria:*** effectiveness, perceived benefit, adherence, personalization, service quality, safety, privacy and security, and accountability. ***Implementation barriers*** include funding (no funding), cost (maintenance), guidelines, and methodology.<br>• **Implementation phase:** The ***goals and tasks*** are to implement and maintain the effective DHI. The ***technical maturity*** refers to an elaborated research prototype that provides the full functionality according to the conceptual model to assess the health impact of individual intervention components. ***Evaluation criteria***: adherence, personalization, perceived benefit, content quality, ethics, service quality, safety, privacy and security, and accountability. ***Implementation barriers*** include Individual resources of end users, expectations (missing accuracy of provided information), usability of technology (technical support, implementation problems), interoperability, human technical support, regional infrastructure, individual characteristics of healthcare providers, negative associations, accessibility, reimbursement, funding (long-term), cost (maintenance), and culture.
**Lee, 2022**	mHealth	Assess apps based on user's needs (healthcare providers,	Experts and potential users	**Scoring matrix (23 items; modification of the MASUN 1.0 and tested with menstrual apps):**<br>App design, widget, alarm, separate features by sex (female or male), app attendance point, indication of menstrual cycle and fertile period (ovulation date), period tracking and symptom recording, menstruation management including dysmenorrhea, PMS management, visualization (graphical chart), visualize menstruation status on the calendar, contraception management, medication, women's health information, healthcare

Reference author, year	DHT category	Scope (target)	Development	Framework/models evaluation: dimensions/criteria/steps for assessment
		researchers, and users)		providers' consultant, women's health checkup, information sharing, user community menu, anonymous user community, information sources, login, unlock mode, privacy policy, and additional features such as easy delivery of women's items (e.g., sanitary napkin and menstrual cup).
**Levine, 2020***	mHealth	Assess quality for DHT informed selection (patients and clinicians)	Experts	**THESIS rating tool derived from the evaluation of available apps (chronic diseases) with 6 domains and 27 categories:**<br>• **Transparency**: cost of app, consent, and accuracy of app store description.<br>• **Health content**: appropriate measurement, appropriate interpretation of data, quality of information, potential for harm, literacy level, and presentation of information.<br>• **Technical content**: software performance/stability, interoperability, bandwidth, and application size.<br>• **Security/Privacy**: protection against theft and viruses, authentication, data sharing, maintenance, signalling of breaches, and anonymization.<br>• **Usability**: installation and setup, functionality, aesthetics, customization/tailoring, ease of use for users with low literacy and numeracy, and availability in multiple languages.<br>• **Subjective**: recommend app, and overall star rating.
**Llorens-Vernet, 2020***	mHealth	Development and assessment of DHT quality (health care providers, developers, patients, and other stakeholders)	Final users, potential patients, health care professionals, and developers or engineers.	**Mobile App Development and Assessment Guide (MAG), with 8 categories grouping 36 important criteria:**<br>• **Usability.** 8 criteria related to user experience.<br>• **Privacy.** 6 criteria related to data protection, compliance with the law, and treatment of users' data.<br>• **Security.** 4 criteria related to criteria related to cybersecurity, encryption mechanisms for the storage and transmission of data, and measures against vulnerabilities.<br>• **Appropriateness and suitability.** 3 criteria related to the adaptation of the app for the benefit of the targeted user.<br>• **Transparency and content.** 5 criteria related to the sharing of information in relation to the development of the app.<br>• **Safety.** 2 criteria related to the identification and prevention of harm to end users.<br>• **Technical support and updates.** 4 criteria related to helping the user to solve problems in using the app.<br>• **Technology**. 4 criteria related to the proper functioning of the app.
**López Seguí, 2018**	General	Framework for recommendation and prescription for apps in a	Experts panel	To be considered in the **Digital Health Platform,** apps must go through an accreditation process, where **evaluation** is assessed through 120 criteria grouped into 4 areas:<br>• **Functionality**: the evaluation of the quality and utility of the app's contents. These are made up of 25 criteria and are given priority over the other 3 areas.

Reference author, year	DHT category	Scope<br><br>(target)	Development	Framework/models evaluation: dimensions/criteria/steps for assessment
		public library (HCPs)		• **Usability and design**: accessibility, user experience, and visual aesthetics.<br>• **Technology**: technological reliability and adaptability of the app in general.<br>• **Security**: guarantee of data security and adherence to data management policies
**Mathews, 2019***	General	Framework for evaluation (all stakeholders)	Experts panel	**Digital Health Scorecard, organised in 4 domains:**<br><br>• **Technical validation:** Assessment of performance when compared to technical gold standard; testing of security features; and testing of interoperability features.<br>• **Clinical validation:** Critical appraisal of evidence supporting whether solution has impact on defined clinical outcomes, comparison to existing clinical gold standard, and real-world testing or simulation performance in target population.<br>• **Usability:** Assessment using standardized usability framework that evaluates performance across basic characteristics (e.g., helpful; effective; learnable; likeable).<br>• **Cost:** Purchase price, resources including time required for training, set-up, implementation, and management of solution, and anticipated cost on impact on clinical outcome of interest.
**Moshi, 2020***	mHealth	HTA evaluation module (regulatory and reimbursement purposes)	Healthcare practitioners, app developers, and policymakers	The following **HTA domains were modified/adapted from traditional HTAs:**<br><br>• **Description and technical characteristics**: Operating systems (i.e. Android, iOS, etc.) and platforms (i.e. smartphone, tablet, smartwatch) of the MMA.<br>• **Current use of technology**: rationale for use (intended purpose, healthcare condition or situation that the MMA addresses, MMA input, algorithm, and output) and potential software updates (post-market software changes that require or not re-evaluation.<br>• **Effectiveness**: accuracy (closeness of the output to the true value to the MMA's output and accuracy measures the effect of software errors on the MMA output), configuration, communication and display, cybersecurity and connectivity, potential software changes (adaptive, corrective, or preventive), precision (degree to which the recurrent measurements input into the MMA generates the same output), and analytical validity (ability to reliably and accurately produce the intentional output from the input data, the algorithm used by the MMA is a recognised standard, MMA accuracy is relative to reference standard, MMA comparable to another software or device that has an association between the software output and a health outcome).<br>• **Safety**: How the MMA output (i.e. information) affects clinical decision making regarding management of a patient's condition.<br>• **Cost-effectiveness**: Considerations of applicability of the system, platform, licensing, attachable hardware, and versions of the MMA to those that would be used in the health system, and unit costs including MMA costs and in-app purchases.<br>• **Organisational aspects**: digital health literacy, responsibility (accreditation that may be needed for professionals to prescribe and/or use the MMA), connectivity (interaction with current health informatics systems such as hospitals and surgeries), and technological evolution (any possible conflicts of interest).

Reference author, year	DHT category	Scope (target)	Development	Framework/models evaluation: dimensions/criteria/steps for assessment
				• **Legal aspects**: Litigation risks to the relevant person(s) associated with the use or recommendation of the MMA for healthcare practitioners, how insurance(s) for all stakeholders (i.e., patients, medical professionals, developers) could be affected through use or recommendation of the MMA, how possible professional registrations could be affected through the use or recommendation of the MMA, clarify which party owns the data related to the MMA, clarity around which party (i.e. manufacturer, medical practitioner who prescribed it) is responsible for the medical advice provided by the MMA, and clarity around which party (i.e. manufacturer, medical practitioner, app developer) is responsible for monitoring and reviewing the patient data entered into the MMA.<br>• **Post-marketing monitoring**: Post-market data that requires a full review of the effectiveness and safety of<br>• an MMA, how the manufacturer plans to monitor the MMA's performance data, how the data collection implemented has the least user burdensome approach to collect the MMA's performance data, how the post-market data could be used to enable or disable new MMA functionalities, how post-market data could affect the MMA's cost-effectiveness, safety, and effectiveness, and how post-market data could affect the ethical, legal, and/or organisational concerns associated with the MMA.<br>• **Social aspects:** How the use of the MMA may affect the patients' caregiver(s), including relationships with medical professionals, how the use of the MMA may affect the users' relationships (i.e., family dynamics, friends, and other relevant social relations), and how the MMA may benefit patient autonomy.
**Nouri, 2018**	mHealth	Framework for evaluation (all stakeholders)	Experts panel	Assessment criteria from a **systematic review reorganized into 7 main classes with 37 subclasses of criteria**:<br><br>• **Design:** suitability of design, aesthetics, appearance, and design consistency.<br>• **Information/Content:** credibility, accuracy, and quality and quantity of information.<br>• **Usability:** ease of use, operability, visibility of system status, user control and freedom, consistency and standards, error prevention, completeness, information needs, flexibility/customizability, competency, style, behaviour, and structure.<br>• **Functionality:** performance, health warnings, feedback, connectivity and interoperability, record, display, guide, remind/alert, and communicate.<br>• **Ethical Issues:** beneficence, non-maleficence, autonomy, justice, and legal obligations.<br>• **Security and Privacy**<br>• **User-perceived value:** subjective quality
**Van Royen, 2023**	Artificial intelligence (AI)	Quality criteria	Experts	**Five quality criteria for AI-based prediction models** in the cardiovascular health domain (probably applicable to other fields):<br><br>• **Complete reporting and reproducibility of results:** Complete reporting should include the detailed description of all steps of the modelling process, including all data preparation steps, all model selection, tuning, recalibration, testing steps, and all results from internal and external validation procedures.

Reference author, year	DHT category	Scope (target)	Development	Framework/models evaluation: dimensions/criteria/steps for assessment
				• **Clear intended clinical use of the AI-based model:** the intended role of the AI-based prediction model in the clinical decision-making process, should be precisely defined to allow for early and careful consideration of the potential clinical consequences of using the model downstream in clinical care.<br>• **Rigorous model validation:** besides internal and external validation techniques, decision curve analysis, health technology assessments, and impact studies (e.g. via randomized clinical trials) can generate valuable information on the clinical benefit and risks of an AI-based prediction model.<br>• **Sufficient sample size for AI model development and validation: c**urrently, there are no calculators available that can be used to do a priori sample size calculations for the development of AI models. However, simulation studies and a posteriori approaches, such as a learning curve approach, may be used to justify the sample size.<br>• **Openness of data and software:** Providing contact details for data and algorithm accessibility requests. For open software, include the code to apply the model in a new setting. Data and software should be publicly available.
**Wagneur, 2022**	mHealth	Assess clinical quality and relevance (designers, investors, hospital clinicians or IT department)	Patients, eHealth experts, and app creators	Development of the multidomain pre-screening **Medical Digital Solution scoring tool, consisting of 26 questions regarding 4 categories:**<br>• **General information about the solution:** type of algorithm used in the solution (no artificial intelligence [AI], no algorithm, intelligible AI, non-intelligible AI, or algorithm >5 years old) and possibility of interaction with the user (yes, with remote monitoring, alerts, information not personalized, with teleconsultation or none)<br>• **Target population of the solution:** Age (<18, 18-64, or >65), number of patients involved in the development (>500, 50-499, 1-49, or 0), impact on hospital organization (simplification, complication, or no impact).<br>• **Clinical relevance of the solution:** validated outcome (user satisfaction, QoL, medico-economic benefit, early diagnosis gain, improved access to acre, improved treatment compliance, reduction of severity, prevention, survival, reduction of emergencies, and less toxicity than reference), level of evidence on clinical assessment (expert advice, retrospective study of ≥300 evaluable patients, applications of national or international recommendations, randomised trial of <200 patients, randomised trial of ≥200 patients, prospective study of ≥200 patients or a meta-analysis, prospective study of ≥200 patients or a meta-analysis vs. nonrandomised control arm in real-life settings, or not based on any studies or expert opinions).<br>• **Information on the solution provider and reimbursement ambition and probability:** medical management by a specialist doctor, by a non-specialist doctor, or no medical direction), development of previous eHealth solutions or medical device with >500 users (yes, 1, 2, or 3 or more, or no), business model (yes, no need), and probability of reimbursement (high, medium, or low).
**Woulfe, 2022**	mHealth	Framework for quality assessment (to	Experts	**MES scale, a modification of the Enlight Suite tool (scale to rate the quality of mHealth and web pages) to improve its international applicability. The MES has 7 sections and 32 evaluating items (5 novel):**

Reference author, year	DHT category	Scope (target)	Development	Framework/models evaluation: dimensions/criteria/steps for assessment
		be internationally applicable)		• **Usability**: navigation, **understandability, errors, timeliness**, learnability, and ease of use.<br>• **Design:** aesthetics, layout, and size.<br>• **Engagement:** concept presentation, interactive, not irritating, targeted/tailored/personalized, and captivating.<br>• **Content:** evidence-based, cultural appropriateness, quality of information provision, clarity about the app purpose, and complete and concise.<br>• **Therapeutic persuasiveness:** call to action, **rewards**, **real data-driven adaptive content**, therapeutic rationale and pathway, ongoing feedback, and expectations and relevance.<br>• **Therapeutic alliance:** acceptance and support, positive therapeutic expectations, and relatability.<br>• **General subjective evaluation:** appropriate features to meet the clinical claim, right mix of ability and motivation, and I like the ap.
